# Prevalence of Antimicrobial Resistance and Association with Patient Outcomes in a Rural Kenyan Hospital

**DOI:** 10.4269/ajtmh.22-0311

**Published:** 2023-05-09

**Authors:** Ian C. Drobish, Immaculate K. Barasa, George Otieno, Moses Odhiambo Osoo, Solomon K. Thuo, Kaya S. Belknap, Arianna McLain Shirk, S. Taylor McClanahan, Elizabeth Irungu, Felix Riunga, Faith Lelei, Kristina E. Rudd, B. Jason Brotherton

**Affiliations:** ^1^ Department of Internal Medicine, Department of Pediatrics, University of California San Diego, San Diego, California;; ^2^ Critical Care Medicine Department, NIH Clinical Center; Bethesda, Maryland;; ^3^ Department of Pediatrics, AIC Kijabe Hospital, Kijabe, Kenya;; ^4^ Department of Internal Medicine, AIC Kijabe Hospital, Kijabe, Kenya;; ^5^ Department of Research, AIC Kijabe Hospital, Kijabe, Kenya;; ^6^ Department of Laboratory Medicine, AIC Kijabe Hospital, Kijabe, Kenya;; ^7^ Department of Outpatient Medicine, AIC Kijabe Hospital, Kijabe, Kenya;; ^8^ Bessemer Pediatrics, Bessemer, Alabama;; ^9^ Department of Pharmacy, AIC Kijabe Hospital, Kijabe, Kenya;; ^10^ Department of Infectious Diseases, Aga Khan University Hospital, Nairobi, Kenya;; ^11^ Department of Family Medicine, AIC Kijabe Hospital, Kijabe, Kenya;; ^12^ Department of Critical Care Medicine, The Clinical Research, Investigation, and Systems Modeling of Acute Illness Center, University of Pittsburgh, Pittsburgh, Pennsylvania

## Abstract

Data on antimicrobial resistance (AMR) and association with outcomes in resource-variable intensive care units (ICU) are lacking. Data currently available are limited to large, urban centers. We attempted to understand this locally through a dual-purpose, retrospective study. Cohort A consisted of adult and pediatric patients who had blood, urine, or cerebrospinal fluid cultures obtained from 2016 to 2020. A total of 3,013 isolates were used to create the Kijabe Hospital’s first antibiogram. Gram-negative organisms were found to be less than 50% susceptible to third- and fourth-generation cephalosporins, 67% susceptible to piperacillin–tazobactam, 87% susceptible to amikacin, and 93% susceptible to meropenem. We then evaluated the association between AMR and clinical characteristics, management, and outcomes among ICU patients (Cohort B). Demographics, vital signs, laboratory results, management data, and outcomes were obtained. Antimicrobial resistance was defined as resistance to one or more antimicrobials. Seventy-six patients were admitted to the ICU with bacteremia during this time. Forty complete paper charts were found for review. Median age was 34 years (interquartile range, 9–51), 26 patients were male (65%), and 28 patients were older than 18 years (70%). Septic shock was the most common diagnosis (*n* = 22, 55%). Six patients had AMR bacteremia; *Escherichia coli* was most common (*n* = 3, 50%). There was not a difference in mortality between patients with AMR versus non-AMR infections (*P* = 0.54). This study found a prevalence of AMR. There was no association between AMR and outcomes among ICU patients. More studies are needed to understand the impact of AMR in resource-variable settings.

## INTRODUCTION

Sepsis is associated with one in five deaths worldwide, and a large burden of this disease falls upon sub-Saharan Africa (SSA).^1^ In addition, prevalence of antimicrobial resistance (AMR) is growing globally and the WHO has declared it one of the 10 most pressing health-care issues.^2^ Data on the prevalence and epidemiology of AMR in resource-limited settings are lacking.^3^^,^^4^ The little we do know comes from larger, urban academic centers, which do not represent where the majority of individuals in these settings live.

Recently, an international study^5^ describing infections among patients in intensive care units (ICUs) in predominantly high-income countries showed that most infections were the result of Gram-negative organisms. This study also noted that infections resulting from resistant Gram-negative organisms were associated independently with a greater risk of death. A recent systematic review^6^ of the etiology and outcomes of sepsis in SSA showed that outcomes were worse in individuals with bloodstream infections compared with those without. That review identified and highlighted the lack of information on AMR in SSA. In addition, in 2014, the WHO identified Africa as one of two regions without established antimicrobial resistance surveillance systems.^7^

Therefore, our objectives were 2-fold. First, to describe for the first time the current landscape of pathogens and resistance through the creation of a local antibiogram in a broad cohort of patients at our rural Kenyan hospital. Second, among a separate cohort of critically ill patients with the greatest exposure to antimicrobials and risk for AMR, to evaluate the association between AMR bloodstream infection and patient characteristics, management, and outcomes. Our hypotheses were that there was a high prevalence of AMR in our patient population, and that those infected with AMR organisms would have increased risk for hospital mortality compared with those who had bacteremia resulting from non-AMR organisms.

## MATERIALS AND METHODS

### Study setting.

Kijabe Hospital (KH) is a 360-bed academic, tertiary care referral center located in rural Kenya. Services available at KH include internal medicine, general surgery, orthopedic surgery, obstetrics and gynecology, and pediatrics. There are dedicated laboratory and phlebotomy teams that provide most services 24 hours per day, 7 days per week.

At the time of this study KH had five ICU beds with five functioning mechanical ventilators, and this single ICU admitted both critically ill adults and children. Admissions to the ICU at KH are a mix of trauma, postsurgical, and general medicine cases. At the time of this study, as is common in many resource-constrained settings, there was no formally trained critical care or infectious disease physician on site. Currently, KH is a training site for a nationally recognized diploma program for clinical officers in emergency and critical care medicine.^8^ Continuous cardiopulmonary monitoring, infusion pumps, and basic laboratory services were available at the facility. Available vasopressors included norepinephrine, dopamine, and epinephrine. Noninvasive ventilation was not available at KH during the time of this study. The antibiotics available at KH are found on the WHO Essential Medications List.^9^ Availability of broader spectrum agents such as piperacillin–tazobactam, ceftazidime, and meropenem was subject to supplier distribution. As is common with many resource-variable settings, the ability to perform laboratory tests is also dependent on the presence of collection supplies, testing reagents, and proper functioning of equipment.

### Study participants.

#### Cohort A.

The data for the antibiogram were collected from February 2016 to September 2020. For the creation of the antibiogram, specimens collected included blood, urine, and cerebrospinal fluid (CSF). These specimens came from both outpatient and inpatient adult and pediatric patients with suspected infections. These specimens were chosen because they are the most commonly obtained specimens at KH. Culture data from 2016 to 2018 were collected retrospectively from a paper spreadsheet housed in the KH laboratory. From 2018 to 2020, the data were collected prospectively in the same manner. No patient records were accessed for the collection of cohort A data.

#### Cohort B.

Cohort B was comprised of patients admitted to the KH ICU from the emergency department, ward transfer, or outside hospital referral. They were admitted by either the medical or surgical services and were included if they had a positive blood culture during that admission. Data for patients admitted to the ICU with bacteremia January 2016 through December 2018 were collected retrospectively from patient paper charts. At the beginning of 2019, KH transitioned from a paper to an electronic medical record system that did not allow for retrospective data collection.

All blood culture results during the study period were reviewed, and patients with at least one positive blood culture result who were admitted to the ICU at any time during the same hospital admission were retained for further analysis. There were no patients with more than one pathogenic organism identified on blood culture during the study period. After identifying all patients with at least one pathogenic organism isolated on blood culture who were admitted to the ICU at any point during that hospital admission, we attempted to retrieve each patient’s paper medical file in the KH Medical Records Department. If and when the paper chart was identified, we abstracted each patient’s demographic, diagnostic, management, and outcome data.

The primary exposure was bacteremia resulting from an AMR organism versus a non-AMR organism. Antimicrobial-resistant organisms were defined as those being resistant to one or more classes of antimicrobial agents.^2^ The primary outcome of interest was outcome of the hospitalization, defined as death, discharge, or transfer to inpatient status at a different medical facility. Secondary outcomes included clinical management variables such as antibiotics given, vasopressor requirement, intravenous fluids administered, and need for mechanical ventilation. In addition, ICU length of stay and duration of hospital stay were also collected.

Vital signs, including Glasgow Coma Scale score, were recorded as close as possible to the time the blood cultures were documented as being collected. To assess severity of illness in patients with suspected infection we calculated quick sequential organ failure assessment (qSOFA) scores using the clinical variables. Admission diagnoses were recorded based on the treating clinician’s documentation. Each patient could have more than one diagnosis, and the percentages were calculated from the total number of diagnoses given.

### Microbiological processes.

The standard practices at KH for the preparation of bacterial cultures during the time of the study were as follows. All blood samples were inoculated in pediatric BACTEC bottles and incubated in a BACTEC BD40 machine (BD Nairobi, Kenya) until bacterial growth was detected or 5 days, whichever came first. If no growth was detected at 5 days, the sample was considered negative. After growth detection, the culture medium was subsequently placed on an agar plate for further incubation. After colony-forming units were noted, Gram staining was performed.

Urine or CSF samples were suspended and plated on one of several types of differential media (e.g., MacConkey agar; cysteine-, lactose-, and electrolyte-deficient agar; Mannitol agar). After colony-forming units were noted, Gram staining was performed.

From 2016 to 2018, gram-negative organisms were speciated further Analytical Profile Index^®^ kits. To determine sensitivity to antimicrobial agents, the Kirby Bauer disk diffusion method was used. Based on Clinical Laboratory and Standards Institute guidelines, zone of growth inhibition was measured with a ruler to determine whether an isolate was sensitive, intermediate, or resistant to a particular antibiotic agent.^10^ Susceptibility testing was subject to disk availability. Starting in 2018, Analytical Profile Index^®^ kits were no longer used, and speciation and susceptibilities were performed using a Vitek^®^ 2 COMPACT machine.

Gram-positive organisms were speciated using stepwise bacterial identification tests (e.g., catalase, oxidase, coagulase tests). After speciation, sensitivities were obtained using the Kirby Bauer disk diffusion method, as just mentioned. From 2018 on, speciation and susceptibility testing for gram-positive organisms were performed using the Vitek^®^ 2 COMPACT machine.

When speciation and susceptibility testing were completed, the results were either entered into a logbook manually by the laboratory staff or a Vitek^®^ 2 COMPACT report was generated. Infection prevention and control staff transcribed the data subsequently into a Microsoft Excel spreadsheet. Using BacLink software, the data from the Excel spreadsheet were converted into a WHONET-compatible format for analysis. Results from 2016 through 2020 were aggregated to achieve the minimum number of isolates (*n *= 30) required for analysis.^11^ At this time, there are no central quality control standards to which laboratories are subjected in Kenya. Recently, a group carried out a convenience sample of 219 laboratory managers at different facilities in Kenya. Of those sampled, the majority of facilities (*n* = 135, 61%) did not perform bacterial culture testing, and even fewer performed antimicrobial susceptible testing (*n* = 37, 17%).^12^

Of note, the local practice at KH was to obtain a single pediatric BACTEC bottle for blood culture analysis, as opposed to two sets, because of the limited supply of culture bottles and cost translated to the patients.

### Statistical analysis.

The WHONET software was used for the creation of the antibiogram using data from cohort A, and percentages were used to express organism sensitivity. Data from cohort B were analyzed using STATA 15 (StataCorp LLC, College Station, TX). For cohort B, we assumed the data were nonparametric because of the small sample size. Categorical values were assigned percentages whereas continuous variables were given median and interquartile range (IQR), which were represented in terms of 25th and 75th percentiles. Because of the aforementioned small sample size, tests of association were performed using χ^2^ or Fisher’s exact tests (if any variable had < 10 observations) and Wilcoxon’s rank sum test. To test for associations of exposure with categorical outcome variables, χ^2^ or Fisher’s exact tests were used. For continuous variables, Wilcoxon’s rank sum test was used. Statistical significance was determined as a *P* < 0.05.

## RESULTS

### Cohort A.

A total of 3,052 distinct isolates were cultures (from either blood, urine, or CSF). There were 1,815 positive blood cultures, 1,156 positive urine cultures, and 81 positive CSF cultures. Ten isolates were omitted prior to the analysis because of erroneous data. Per antibiogram standards, only one isolate per patient was used in each of the analyses.^11^ After removing those organisms with less than 30 total isolates, the number reduced to 3,013. Table 1 represents the completed antibiogram and Table 2 lists the most common species identified and their trends by year. Of the 3,013 isolates, 1,534 (51%) were coagulase-negative *Staphylococcus* (CoNS), 721 (24%) were *Escherichia coli*, 465 (15%) were *Klebsiella *sp., and 110 (3.7%) were *Staphylococcus aureus*.

**Table 1:** AIC Kijabe Hospital antibiogram

AIC Kijabe Hospital antibiogram	Organism	No. of isolates	Amikacin	Amoxicillin–clavulanic acid	Aztreonam	Cefazolin	Cefepime	Cefotaxime	Cefoxitin	Ceftazidime	Ceftriaxone	Cefuroxime	Chloramphenicol	Ciprofloxacin	Clindamycin	Cloxacillin	Gentamicin	Meropenem	Nitrofurantoin	Piperacillin–tazobactam	Trimethoprim–sulfamethoxazole	Vancomycin
Gram negative	*Acinetobacter baumannii*	50	72	–	–	–	46	–	–	33	29	–	–	51	–	–	65	57	–	44	31	–
	*Enterobacter cloacae*	63	82	–	–	–	56	–	–	35	31	–	63	60	–	–	46	90	54	63	25	–
	*Escherichia coli*	721	88	57	49	–	52	49	81	48	49	47	78	55	–	–	70	96	85	75	19	–
	*Klebsiella *spp.	465	87	35	38	–	32	29	82	27	29	26	59	61	–	–	42	94	53	52	25	–
	*Pseudomonas aeruginosa*	39	82	–	–	–	70	–	–	65	–	–	–	76	–	–	87	82	–	74	–	–
	*Serratia fonticola*	31	97	54	–	–	52	–	75	52	29	29	–	30	–	–	–	97	84	65	30	–
Gram positive	*Staphylococcus aureus*	110	–	–	–	87	–	–	–	–	–	–	90	–	66	91	–	–	–	–	32	70
	*Staphylococcus*, coagulase negative	1,534	–	–	–	70	–	–	–	–	–	–	87	–	57	69	–	–	–	–	27	80

This is aggregated culture data from blood, urine, and CSF for the years 2016 to 2020. The minimum of 30 isolates were used to create the antibiogram. Numbers are expressed as percent sensitive.

**Table 2:** Most common bacterial isolates at AIC Kijabe Hospital by year

Organism	No. of isolates (%)	2016*	2017	2018	2019	2020†
*Staphylococcus*, coagulase negative‡	1,531 (51)	360	394	471	191	115
*Escherichia coli*	720 (24)	101	148	199	203	69
*Klebsiella *spp.	464 (15)	79	108	135	94	48
*Staphylococcus aureus*	110 (4)	36	23	31	12	8
*Enterobacter cloacae*	63 (2)	14	17	15	10	7
*Acinetobacter baumannii*	50 (2)	8	13	16	9	4
*Pseudomonas aeruginosa*	39 (1)	6	14	11	6	2
*Serratia fonticola*	31 (1)	–	5	14	6	6

* Data from 2016 started in February.

† Data from 2020 is incomplete (January–September) because of the supply-chain burden induced by the COVID-19 pandemic.

‡ Not considered pathogenic at AIC Kijabe Hospital outside of neonates or patients with central venous catheterization. The total number of isolates (*N *= 3,008) reflects the isolates cultured with more than the requisite 30 isolates per organism to be used to create an antibiogram.

**Figure 1.**
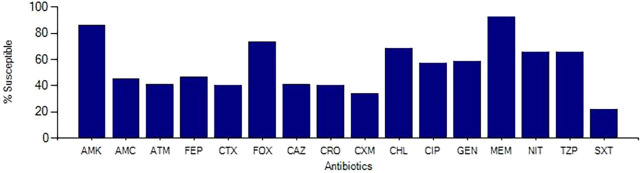
Aggregate susceptibility of gram-negative organisms. All blood, urine, and cerebrospinal fluid samples with gram-negative rod isolates identified were included and analyzed in aggregate. AMC = amoxicillin–clavulanic acid; AMK = amikacin; ATM = aztreonam; CAZ = ceftazidime; CHL = chloramphenicol; CIP = ciprofloxacin; CRO = ceftriaxone; CTX = cefotaxime; CXM = cefuroxime; FEP = cefepime; FOX = cefoxitin; GEN = gentamicin; GNR = gram negative rod; MEM = meropenem; NIT = nitrofurantoin; SXT = trimethoprim–sulfamethoxazole; TZP = piperacillin–tazobactam.

**Figure 2.**
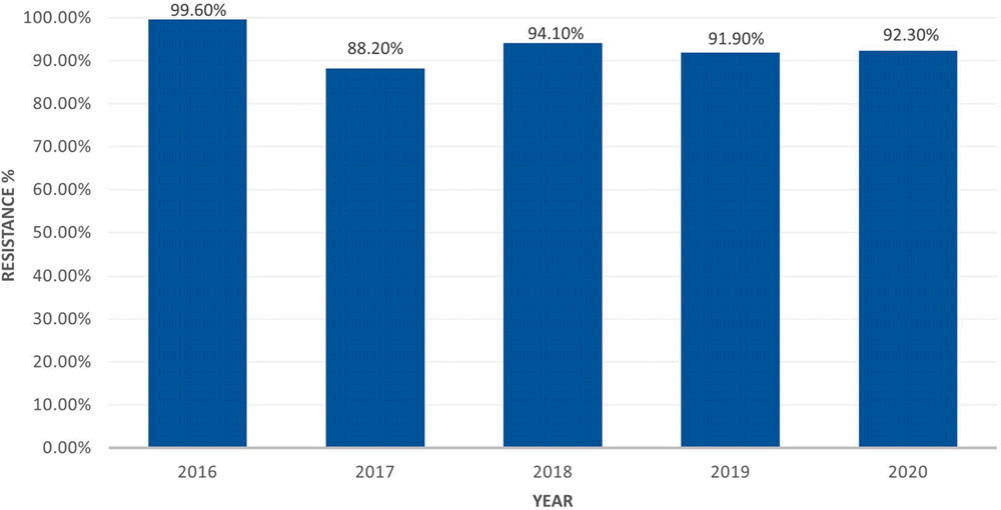
Percent of gram-negative antimicrobial-resistant organisms by year. All blood, urine, and cerebrospinal fluid samples with gram-negative rod isolates identified were included. This is expressed as a percentage of the number of antimicrobial-resistant gram-negative organisms divided by the total number of gram-negative organisms isolated.

Aggregate susceptibility patterns of gram-negative organisms are shown in Figure 1. Collectively, gram-negative organisms were found to be less than 50% susceptible to third- and fourth-generation cephalosporins (42.0%, ceftazidime; 41%, ceftriaxone; 42%, cefotaxime; and 48%, cefepime), whereas only 67% were susceptible to piperacillin–tazobactam, 87% were susceptible to amikacin, and 93% were susceptible to meropenem. Evaluating specific gram-negative organisms, *E. coli* was found to be susceptible to third- and fourth-generation cephalosporins at greater rates than other gram-negative organisms (48%, ceftazidime; 49%, ceftriaxone; 49%, cefotaxime; and 53%, cefepime), and 75% were susceptible to piperacillin–tazobactam, 88% susceptible to amikacin, and 96% susceptible to meropenem. *Klebsiella* species were less sensitive (27%, ceftazidime; 29%, ceftriaxone; 29%, cefotaxime; and 32%, cefepime), with 52% susceptible to piperacillin–tazobactam, 87% susceptible to amikacin, and 94% susceptible to meropenem. *Acinetobacter baumannii* was found to have lower rates of susceptibility for all antibiotics tested (29–33% for third-generation cephalosporins), 46% susceptible to cefepime, 44% susceptible to piperacillin–tazobactam, 72% susceptible to amikacin, and 57% susceptible to meropenem. Table 2 shows the susceptibility profile for the most common organisms is listed. The percentage of all gram-negative organisms identified as resistant by year can be seen in Figure 2. This was consistent each year of the study period.

Regarding gram-positive organisms, *S. aureus* was 32% susceptible to trimethoprim–sulfamethoxazole, 66% susceptible to clindamycin, 87% susceptible to cefazolin, and 91% susceptible to cloxacillin. Testing against oxacillin or methicillin was not performed routinely at KH.

### Cohort B.

During the cohort B study period, 76 patients admitted to the ICU had positive blood cultures during the same admission. Seventeen patients (22%) were found to have gram-negative infections, and 13 of these (76%) were found to be the result of an AMR organism (Supplemental Figure 1). Despite a thorough search, only 40 complete paper files were found from the original 76. These 40 patients were retained for further analysis, and their characteristics, management, and outcomes are described below.

Median patient age was 34 years (IQR, 9–51 years), and the majority were male (*n* = 26, 65%) (Table 3). Of the 40 patients included in the study, 28 (70%) were 18 years or older and 12 (30%) were younger than 18 years. Only one pediatric patient was found to have an AMR infection. The most common diagnosis was septic shock (*n* = 22, 55%), followed by acute kidney injury (*n* = 16, 40%), respiratory failure (*n* = 14, 35%), and pneumonia (*n* = 7, 18%). The overall hospital mortality was 55% (*n* = 22) (Table 3). For reference, mean overall mortality during the cohort B study period for all patients admitted to the KH ICU was 30%. There was not an increased risk of death found between those with an AMR bloodstream infection versus those with bacteremia resulting from a non-AMR organism [*n* = 4 (67%) versus *n* = 18 (53%), *P* = 0.54].

**Table 3:** Baseline characteristics, hospital outcomes, physiological and laboratory values, and management of Cohort B

Variable	All (*n* = 40)	AMR (*n* = 6)	Non-AMR (*n *= 34)	*P* value
Age, years; median (IQR)	34 (9–51)	54 (45–63)	30 (4–47)	–
Age, years; *n* (%)				
0–5	9 (23)	0 (0)	9 (23)	–
6–17	3 (7.5)	1 (17)	2 (5.8)	–
≥ 18	28 (70)	5 (83)	23 (68)	–
Female, *n* (%)	14 (35)	3 (50)	11 (32)	–
Admission diagnosis,* *n* (%)				
Septic shock	22 (55)	4 (67)	18 (53)	–
Acute kidney injury	16 (40)	5 (83)	11 (32)	–
Respiratory failure	14 (35)	2 (33)	12 (35)	–
Pneumonia	7 (18)	1 (17)	6 (18)	–
Bacteria, *n* (%)				
*Escherichia coli*	4 (10)	3 (50)	1 (3)	–
*Klebsiella* spp.	2 (5)	2 (33)	0 (0)	–
*Enterobacter* spp.	1 (2.5)	1 (17)	0 (0)	–
*Citrobacter* spp.	1 (2.5)	0 (0)	1 (3)	–
*Staphylococcus aureus*	3 (7.5)	0 (0)	3 (8.8)	–
Coagulase-negative *Staphylococcus*	29 (73)	0 (0)	29 (85)	–
ICU LOS, days; median (IQR)	4 (1–9)	2 (1–5)	4 (1–9)	–
Hospital LOS, days; median (IQR)	7 (2–16)	3.5 (1–10)	8 (3–18)	–
Death, *n *(%)	22 (55)	4 (67)	18 (53)	0.54
Discharge, *n *(%)	14 (35)	1 (17)	13 (38)	0.31
Transfer, *n *(%)	4 (10)	1 (17)	3 (8.8)	0.56
Systolic blood pressure, mm Hg; median (IQR)	103 (84–121)	101 (84–115)	103 (84–124)	0.94
Pulse, beats/minute; median (IQR)	128 (111–141)	135 (102–144)	127 (111–140)	0.61
Respiratory rate, breaths/minute; median (IQR)	25 (20–39)	22 (20–25)	26 (20–40)	0.25
Peripheral oxygen saturation, median (IQR)	92 (89–96)	91 (85–100)	92 (89–96)	0.97
GCS score, median (IQR)	9 (4–14)	9 (4–11)	9 (4–14)	0.86
qSOFA score, median (IQR)	2 (1–2)	2 (1–2)	2 (1–2)	0.68
qSOFA score ≥ 1, *n* (%)	40 (100)	6 (100)	34 (100)	–
Laboratory values, median (IQR)				
Hemoglobin,† g/dL	13 (10–15)	10 (8.1–11)	13 (11–15)	0.34
WBC,‡ ×10^3^/μL	14 (10–23)	10 (5.3–30)	15 (11–23)	0.34
Platelets,§ ×10^3^/μL	220 (162–350)	191 (162–487)	242 (160–350)	0.34
Creatinine,‖ mg/dL	1.4 (1–2.4)	2.2 (1.3–3)	1.2 (1–2.2)	0.36
Mechanical ventilation, *n* (%)	32 (80)	5 (83)	28 (82)	0.83
Vasopressor administered, *n* (%)				
24 hours before culture¶	11 (28)	4 (67)	7 (21)	0.04
24 hours after culture#	22 (55)	4 (67)	18 (53)	0.67
Volume of IVF administered, mL; median (IQR)				
24 hours before culture**	1,675 (750–2,465)	1,700 (575–2,725)	1,675 (900–2,293)	0.89
24 hours after culture††	2,430 (647–3,000)	3,300 (2,430–3,500)	2,125 (550–2,880)	0.07
Initial antibiotic administered, *n* (%)				
Ceftriaxone	20 (50)	4 (67)	2 (33)	0.38
Piperacillin/tazobactam	16 (40)	2 (33)	4 (67)	0.72
Metronidazole	13 (33)	4 (67)	2 (33)	0.06
Meropenem	10 (25)	1 (17)	5 (83)	0.61

AMR = antimicrobial resistance; GCS = Glasgow Coma Scale; ICU = intensive care unit; IQR = interquartile range; IVF = intravenous fluid; LOS = length of stay; qSOFA = quick sequential organ failure assessment; WBC = white blood cell. Antibiotic percentages were calculated using the denominator of 40 patients. Multiple antibiotics could be given to a single patient, and regimens could be changed based upon culture results. Therefore, the number of antibiotics given will not sum to 100%.

* Patients were given more than one diagnosis. These are the most common. Percentages were calculated by using the denominator of each column and do not add up to 100%. Discharge from hospital was considered discharge to home. Transfer was considered transfer to another hospital facility. Physiological variables were collected at ICU admission.

† A denominator of 39 is different for each variable based on the data available from the paper chart review.

‡ A denominator of 37 is different for each variable based on the data available from the paper chart review.

§ A denominator of 37 is different for each variable based on the data available from the paper chart review.

‖ A denominator of 39 is different for each variable based on the data available from the paper chart review.

¶ A denominator of 39 is different for each variable based on the data available from the chart review.

# A denominator of 40 is different for each variable based on the data available from the chart review.

** A denominator of 20 is different for each variable based on the data available from the chart review.

†† A denominator of 29 is different for each variable based on the data available from the chart review.

Coagulase-negative *Staphylococcus* (CoNS) was the most common bacteria isolated within this cohort (*n* = 29, 73%) but is considered nonpathogenic at KH among non-neonates or individuals without central venous catheters (CVCs). At the time of our study, neither of these patient groups were represented in the cohort. *Escherichia coli* was the most common gram-negative organism (*n* = 4, 50%) and was also the most common AMR organism (*n* = 3, 50%). Hospital length of stay [3.5 days (IQR, 1–10 days) versus 8 days (IQR, 3–18 days), *P* = 0.47] and ICU length of stay [2 days (IQR, 1–5 days) versus 4 days (IQR, 1–9 days), *P* = 0.37] were not statistically shorter among AMR patients compared with those with a non-AMR bloodstream infection.

Vital signs at the time of ICU admission did not differ significantly between patients with AMR and non-AMR bacteremia (Table 3). However, the entire cohort was severely ill. Among cohort B, median systolic blood pressure was 103 mm Hg (IQR, 84–121 mm Hg), and median heart rate was 128 beats/minute (IQR, 111–141 beats/minute). In addition, most patients were tachypneic and encephalopathic, with a median respiratory rate of 25 breaths/minute (IQR, 20–39 breaths/minute) and median Glasgow Coma Scale score of 9 points (IQR, 4–14 points). Quick sequential organ failure assessment scores did not differ between cohorts (median score, 2 points; IQR, 1–2 points; for both cohorts; *P* = 0.68). All 40 patients in the cohort had a qSOFA score ≥ 1 point. Last, there were no appreciable differences in laboratory values by AMR status.

Eighty percent (*n* = 32) of individuals in this cohort required mechanical ventilation, but there was no difference between those with AMR versus non-AMR bacteremia [*n* = 5 (83%) versus *n* = 28 (82%), *P* = 0.85] (Table 3). Significantly more patients with AMR infections required vasopressors prior to acquisition of blood culture compared with those with non-AMR infections [*n* = 4 (67%) versus *n* = 7 (21%), *P* = 0.04]. However, this finding dissipated during the 24-hour period after cultures were obtained [*n* = 4 (67%) versus *n* = 18 (53%), *P* = 0.67]. The two groups received similar volumes of intravenous fluid [1,700 mL (IQR, 575–2,725 mL) versus 1,675 mL (IQR, 900–2,293 mL), *P* = 0.89].

All patients in this cohort had suspected infection and were started empirically on antibiotics. The most common initial antibiotic given was ceftriaxone (*n* = 20, 50%). Among those with an AMR blood culture, ceftriaxone and metronidazole were given to 67% (*n* = 4). Piperacillin–tazobactam was given to 16 patients (40%). When culture data became available, two patients with AMR cultures were found to have been started on incongruent antimicrobial therapy.

## DISCUSSION

In our study, the multidisciplinary team at KH set out to establish the prevalence of AMR with the creation of an antibiogram, while also conducting an in-depth review of AMR versus non-AMR bacteremia in the most vulnerable patients in the hospital: those admitted to the ICU. Creation of the antibiogram out of the larger cohort fills a gap in the current knowledge of AMR in resource-variable settings. Our antibiogram findings are congruent with what has been found in large urban settings in East and West Africa.^3^^,^^7^^,^^13^ It also adds to the data that was missing from Kenya for the WHO prioritization study.^14^

There was a significant prevalence of CoNS noted in our culture data. At KH, this is not considered pathogenic outside of neonates or those with CVCs. However at KH, because of a number of factors, CVCs are rarely used. Eliminating CoNS-positive blood cultures isolated outside of the neonatal population reduced the total CoNS number to 466, making the largest percentage of pathogens gram-negative organisms. This finding of gram-negative organisms being the most common pathogen in the region has been described elsewhere.^7^ The largest percentage of AMR organisms were also gram negative regardless of specimen source.^7^^,^^15^^,^^16^ Although the specific pathogens from these studies vary from those isolated at KH, the overarching theme of resistant gram-negative organisms remains.

For our cohort of ICU patients with bacteremia, although our hypothesis that AMR would be associated with worse outcomes was rejected, we did find several observations that are consistent with previous publications. First, as Rudd et al.^1^ found, sepsis and septic shock are indeed common and burdensome at KH. Similarly, after eliminating blood cultures considered to be contaminants, we found that the majority of bloodstream infections were the result of gram-negative organisms, and a large percentage of those were multidrug resistant.^3^^,^^5^ The majority of multidrug-resistant infections occurred in the adult population compared with pediatric.^7^^,^^17^ The overall mortality of this cohort was high, as has been shown previously.^17^

Unlike previous studies, we did not find an increased risk of mortality for those with an AMR infection compared with those without. This could have possibly been the result of several factors. Most notably, although we included all patients admitted to the ICU who had a positive blood culture during the same hospital admission, we had a small sample size. The number of patients included were limited by the number of paper medical records that were able to be retrieved. The most common gram-negative organism speciated was *E. coli*, which is consistent with a study conducted in Kenya at a more highly resourced hospital in the capital of Nairobi.^18^ In that particular study, the authors noted high resistance in gram-negative organisms and low resistance of *S. aureus*, similar to our findings.

Ceftriaxone is on the WHO’s watch group list as an agent that has high resistance potential.^9^ Its broad use as a first-line agent at KH and in SSA is likely one of the key factors for why there is such high resistance. The evidence presented by the antibiogram as well as our small ICU cohort makes it apparent that changes to our empirical treatment practices are warranted. Like many institutions in this context, we are limited in our formulary. A next step for KH may be to use piperacillin–tazobactam, but as the antibiogram has identified, the most commonly cultured gram-negative organism is only 75% susceptible. This could possibly be overcome by using extended intravenous infusions.^15^^,^^16^

In addition, the percentage of carbapenem-resistant organisms, especially *Acinetobacter*, is of significant concern. *Acinetobacter* and *Enterobacteriaceae* have been identified as critical priority pathogens by the WHO that require special attention in addressing their effect on patients in low- and middle-income countries.^14^ An AMR surveillance and genetic sequencing study^14^ from the regional referral center in Nairobi identified 11 different carbapenemase genes in various gram-negative pathogens, suggesting that the presence of carbapenem resistance is more prevalent in Kenya than previously thought. Currently, we are unable to carry out this level of testing at KH, but partnering with the national laboratory or the aforementioned referral center is of future interest.

Other future directions from this study will be to expand the already formed infection prevention and control team into an antimicrobial stewardship team to help improve hospital-wide education and uptake of best practices.^19^ In addition, given the antibiogram is a static measure of resistance patterns of a particular point in time, KH will need to repeat it to continue to monitor these patterns for increasing rates of resistance. Evaluation of AMR trends will have to be viewed in light of the COVID-19 pandemic and the disruption caused to regular hospital functions and admissions. Revisiting Table 2, a sharp drop-off in the number of isolates in 2020 can be seen. As mentioned, this is likely a result of a multitude of factors, not the least of which were those external to KH and Kenya as a whole. In addition, it will be imperative to monitor for worsening or emerging resistance patterns resulting from antimicrobial overprescribing practices that occurred during the COVID-19 pandemic.^20^

Furthermore, restricting the use of broad-spectrum antimicrobials, such as meropenem, at KH will be necessary to thwart selection of more resistant organisms. Not only will a biannual antibiogram be useful in guiding the selection of antibiotic agents, but also it will help identify the areas where quality improvement initiatives can be implemented. For example, moving from identification to determining AMR’s broader impact on patient outcomes will be an important variable to track.

From a regional approach, knowledge translation of our experience to similarly staffed and resourced facilities will be needed to expand the local understanding of AMR further. Developing collaborative relationships with facilities that are both ahead and behind us in these efforts can enhance local knowledge and capacity.

There are several limitations to our study. First, this study was conducted at a single center. Second, there was high laboratory staff turnover during the study period, along with changes in the microbiological equipment and availability of materials. This may have led to varying practices in culture preparation, speciation, and, ultimately, in susceptibility testing. Third, the high rate of CoNS could suggest contamination and the need for improved collection and preparation technique. Not knowing the total number of cultures obtained over the study time eliminated the possibility of determining an accurate contamination rate. Keeping track of this denominator moving forward will allow KH to compare its rate against accepted standards. Next, the inability to determine which infections are community acquired versus hospital acquired limits our ability to make quality improvements in these areas. The low sensitivity of the AMR definition may have overselected for the organisms in question. However, in the resource-constrained setting, waiting until organisms are resistant to three or more organisms is not a tenable option, because there are limited therapeutic choices available.^21^ As with many facilities in resource-variable settings, cost of tests can be a limitation. Having to pay for a test before it can be performed can lead clinicians to err on the side of treatment over investigation, thus limiting the number of patients who have cultures acquired, and may even cause those performed to be false negative as a result of pretreatment. Last, ease of access to antibiotics without a prescription increases the likelihood of AMR and lessens the ability to obtain accurate culture data.^22^^,^^23^

For our ICU cohort, in addition to what has already been mentioned, we were limited by the small number of complete medical files. Furthermore, the practice of obtaining a single bottle (not a single set) for culture purposes at KH during the study period may have limited our ability to capture pathogenic organisms. The limited number of ICU beds available in Kenya and in KH compared with the number of critically ill patients can make it difficult for clinicians to identify patients that may benefit from these precious resources. This could have created an unintended selection bias of severely ill patients in the cohort.

A major strength of the study is that this is a first in a rural Kenyan hospital, likely representing AMR in the communities where the majority of Kenyans reside. Additional strengths are the multidisciplinary team approach, which included laboratory staff, infection control nurses, pharmacists, and several physicians from various departments. Also, this study represents a real-world scenario for many institutions in settings such as KH, where many dynamic factors are at play. For example, missing data, high staff turnover, variability in the presence of equipment and reagents, ability to maintain equipment in functioning condition, and the collateral effects of a global pandemic are all variables that can affect the consistency of microbiological and patient outcome data. Moreover, the constrained resources directly affecting allocation of care make this a generalizable study to many settings, including those that are resource variable. Furthermore, for our ICU cohort, the combined adult and pediatric ICU is likely more common than distinct units found in high-income settings.

## CONCLUSION

It remains clear that AMR is a burgeoning threat worldwide. Our study has shown what many have suspected: AMR is, indeed, present in rural areas that have previously been left out of epidemiological investigations. Although there was not a clear clinical impact of AMR in our ICU cohort, one could posit that the small sample size precluded any such detection. More studies are needed on local and international scales to continue to follow the breadth of this problem. This study points to the critical necessity that future investigations must include patients in rural and resource-variable settings.

## Financial Disclosure

Financial support: K. E. R. is supported by the NIH/National Institute of General Medical Sciences (K23GM141463).

## Supplemental Materials


Supplemental materials
