# Supplementary material for: Prevalence of Antimicrobial Resistance and Association with Patient Outcomes in a Rural Kenyan Hospital
Source: Am J Trop Med Hyg. 2023 May 9;108(6):1227–34. doi: 10.4269/ajtmh.22-0311 (PMC10540121; doi:10.4269/ajtmh.22-0311)
Supplement: Supplementary file 1 [file tpmd220311.SD1.pdf]

e-supplemental Figure 1

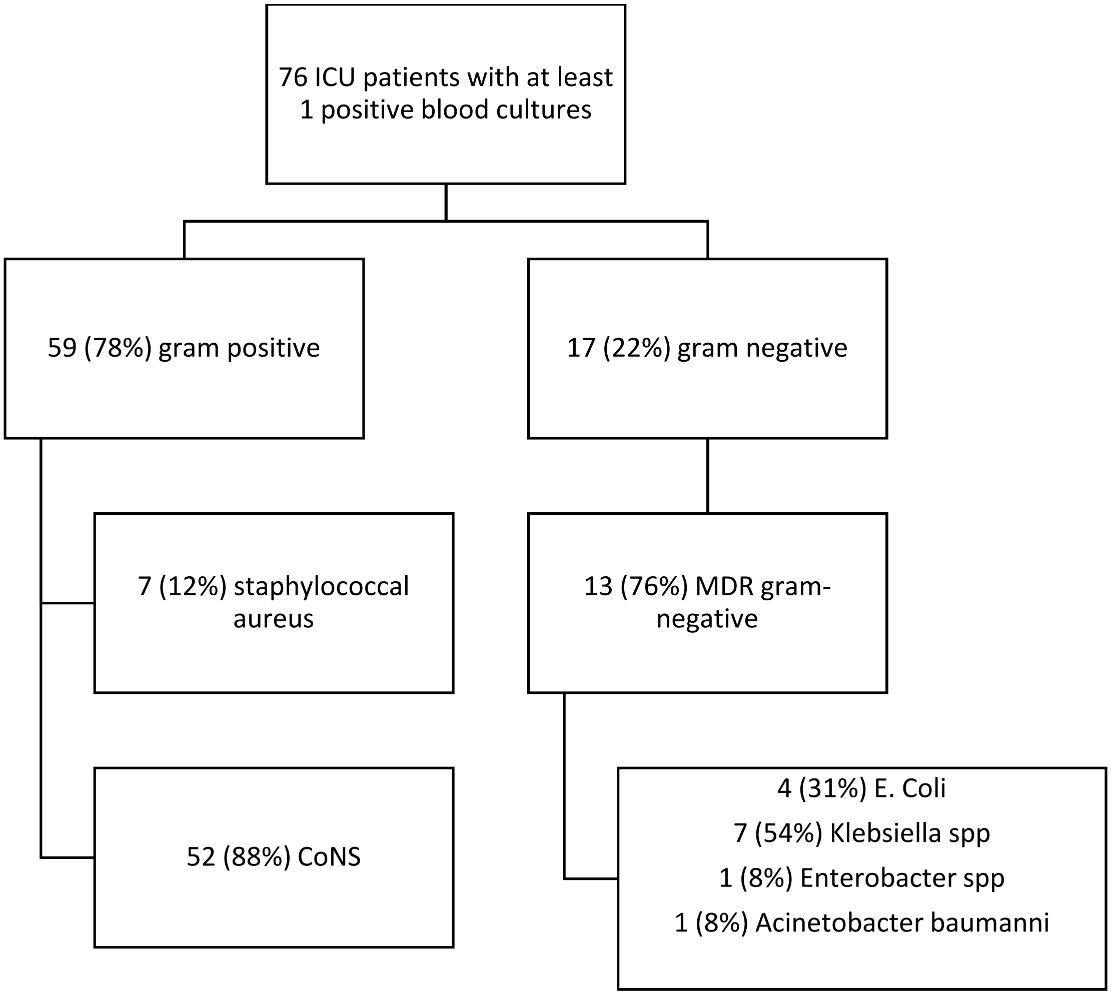

Seventy-six blood culture from ICU patients during study time period. 59 (78%) were gram positive, with 52 (88%) being CoNS. CoNS is considered non-pathogenic at KH in non-neonates and those without central venous cannulation. Seventeen cultures (22%) were due to gram negative organisms with 13 (76%) of these being MDR. MDR was defined as resistance to one or more class of antibiotics. ICU, intensive care unit; MDR, multidrug resistant; CoNS, coagulase negative staphylococcus
